# Structural Similarities and Differences between Amyloidogenic and Non-Amyloidogenic Islet Amyloid Polypeptide (IAPP) Sequences and Implications for the Dual Physiological and Pathological Activities of These Peptides

**DOI:** 10.1371/journal.pcbi.1003211

**Published:** 2013-08-29

**Authors:** Chun Wu, Joan-Emma Shea

**Affiliations:** 1 Department of Chemistry and Biochemistry, University of California, Santa Barbara, Santa Barbara, California, United States of America; 2 Department of Physics, University of California, Santa Barbara, Santa Barbara, California, United States of America; Stanford University, United States of America

## Abstract

IAPP, a 37 amino-acid peptide hormone belonging to the calcitonin family, is an intrinsically disordered protein that is coexpressed and cosecreted along with insulin by pancreatic islet β-cells in response to meals. IAPP plays a physiological role in glucose regulation; however, in certain species, IAPP can aggregate and this process is linked to β-cell death and Type II Diabetes. Using replica exchange molecular dynamics with extensive sampling (16 replicas per sequence and 600 ns per replica), we investigate the structure of the monomeric state of two species of aggregating peptides (human and cat IAPP) and two species of non-aggregating peptides (pig and rat IAPP). Our simulations reveal that the pig and rat conformations are very similar, and consist of helix-coil and helix-hairpin conformations. The aggregating sequences, on the other hand, populate the same helix-coil and helix-hairpin conformations as the non-aggregating sequence, but, in addition, populate a hairpin structure. Our exhaustive simulations, coupled with available peptide-activity data, leads us to a structure-activity relationship (SAR) in which we propose that the functional role of IAPP is carried out by the helix-coil conformation, a structure common to both aggregating and non-aggregating species. The pathological role of this peptide may have multiple origins, including the interaction of the helical elements with membranes. Nonetheless, our simulations suggest that the hairpin structure, only observed in the aggregating species, might be linked to the pathological role of this peptide, either as a direct precursor to amyloid fibrils, or as part of a cylindrin type of toxic oligomer. We further propose that the helix-hairpin fold is also a possible aggregation prone conformation that would lead normally non-aggregating variants of IAPP to form fibrils under conditions where an external perturbation is applied. The SAR relationship is used to suggest the rational design of therapeutics for treating diabetes.

## Author Summary

IAPP, a 37 amino-acid peptide hormone belonging to the calcitonin family, is an intrinsically disordered peptide produced along with insulin by pancreatic islet β-cells in response to meals. In its functional form, IAPP acts as a synergic partner of insulin to reduce blood glucose. IAPP can, however, also play a pathological role, contributing to Type II diabetes (T2D). Knowledge of the structural nature of the physiological and pathological forms of IAPP will facilitate the rational design of novel drugs for therapeutic treatment of T2D. However, because IAPP does not fold to a single structure, but rather co-exists between multiple functional (and toxic) structures, it is extremely challenging for experimental methods to gain detailed structural information. Using a computational approach, we were able to obtain detailed structures of four IAPP variants and propose a novel structural hypothesis for the two opposing roles of this peptide.

## Introduction

The Islet Amyloid Polypeptide/IAPP (also known as amylin) is coexpressed and cosecreted with insulin by pancreatic islet β-cells [1]–[3] and acts as a synergistic partner of insulin to limit after-meal glucose excursions [4]. IAPP belongs to the calcitonin (CT) family of peptides (See Table S1 in Text S1 ) [5]–[7]. The CT peptides function as hormones and are distributed in various peripheral tissues (the endocrine pancreas in the case of IAPP), and play important biological roles including reducing nutrient intake (IAPP), decreasing bond resorption (Calcitonin) and vasodilatation (CGRP/calcitonin gene-related peptide). These functions are fulfilled through hormone-receptor agonism in which the CT peptide binds to a signal transduction membrane protein complex and thereby induces cell response in peripheral tissues. In the particular case of IAPP, this peptide binds to multiple amylin-specific (AMY) receptor complexes [1], [7]–[10]. CT peptides show strong sequence homology in their two terminal regions (residues 1–19 and 30–37), and shares three features: [7] an N-terminal disulfide bond, an N-terminal amphipathic region, and C-terminal amidation.

**Figure 1:**
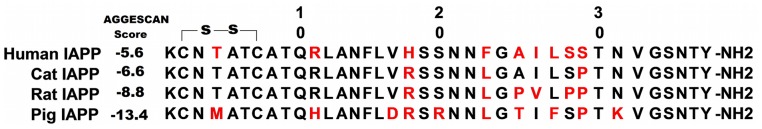
The four IAPP variants listed with aggregation propensities (Na^4^vSS values from AGGRESCAN server). The residues that differ from those of hIAPP are mainly located between residues 4–31 (see residues in red in the sequences). While human and cat IAPP has strong aggregation tendency contributing to T2D, rat and pig IAPP doesn't aggregate under normal conditions and is not toxic to β-cells.

In addition to playing an important physiological role as a CT hormone, IAPP can also play a pathological role. Indeed, the 37-residue long human form of IAPP is the major protein constituent of pancreatic islet amyloid deposits found in 95% of Type 2 Diabetes (T2D) patients [11]–[14]. Interestingly, the IAPP peptide is found in a number of animal species, with a few point mutation differences, yet not all of these animals develop T2D. The development of T2D appears to be directly linked with the inherent aggregation propensity of the peptide. A recent bioinformatics study [15] ranked the aggregation propensities of the IAPP variants using the AGGRESCAN program [16], with the pig sequence emerging as the least aggregation prone and the puffer fish as the most aggregation prone. Figure 1 lists the sequences of four IAPP species that we will consider in this study, and their aggregation propensities. Human and Cat (as well as Monkey and Dog) IAPPs all have high aggregation propensities, and all these species can develop T2D. Tellingly, species (such as rodents and pigs) that are well-known to tolerate excessive food intake without obvious health ramifications, have low aggregation propensities and are not known to develop Type II Diabetes. Transgenic rats, on the other hand, possessing the human variant of IAPP, spontaneously develop T2D when placed on a high calorie, sedentary diet [13], [17]–[21]. In all IAPP variants (Figure 1), the two terminal parts are conserved (namely residue 1–16 and residue 30–37, which we will refer to as “conserved region I and II”, respectively). These are the very regions that are conserved in all CT family peptides and play important biological functions, with conserved region I activating the receptor and conserved region II binding in an antagonistic manner [22]. The mutations that differentiate the different IAPP forms occur in the middle region (residues 18–29, hereafter referred to as “the mutation region”) and are responsible for the different aggregation propensities. The importance of this mid-region in governing aggregation is further highlighted by a familial form of T2D found in Japan that involves a single point mutation in this middle region (S20G). The S20G mutant aggregates more rapidly than its human wild type hIAPP counterpart [23], [24], and leads to β-cell death and early onset T2D [25], [26].

**Figure 2:**
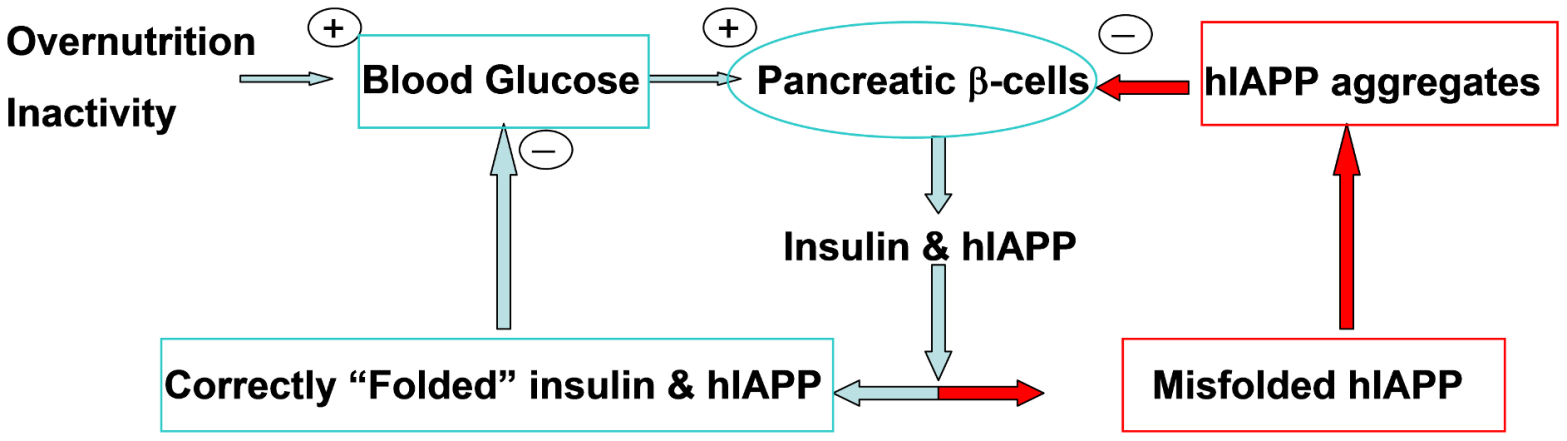
Two opposite roles of hIAPP in T2D. In T2D, over-nutrition and inactivity lead to a high demand for pancreatic β-cells to produce insulin and hIAPP, two hormones that play a critical role in lowering blood glucose. Whereas correctly folded insulin and hIAPP function to reduce blood glucose (left loop), the misfolded form of hIAPP is amyloidogenic and toxic to the β-cells (right loop). +: increase −: decrease.

The above observations suggest a link between IAPP aggregation and the β-cell apoptosis occurring in T2D. Further support for a toxic role of IAPP aggregates include 1) the observation that human hIAPP amyloids play a deleterious role in transplanted islet tissue [27]–[30] and that aggregates of synthetic hIAPP induce apoptotic β-cell death in vitro [31]–[33]. 2) The recent experimental observation of two parallel pathways leading to β-cell apoptosis by hIAPP: one involving extrinsic death signals triggered by extracellular hIAPP aggregates [34], [35], and an intrinsic endoplasmic reticulum (ER) stress pathway linked to the presence of intracellular hIAPP aggregates [36]–[40]. **3)** Experimental evidence of a membrane-damaging effect of hIAPP aggregates leading to β-cell dysfunction [41], [42]. **4)** The fact that blocking aggregation of hIAPP through interaction with non-aggregating hIAPP mutants [43]–[46] as well as small molecules (e.g. EGCG [47], tetracycline [48] and resveratrol [49]) can reduce hIAPP-induced toxicity. A summary of a putative scheme embodying the functional and pathological roles of IAPP is given in Figure 2.

The fact that some IAPP variants aggregate while others do not raises the question as to whether the small sequence differences between species (Figure 1) could lead to structural differences in the monomeric forms of these peptides that would lead one species to favor aggregate prone conformations. This question has been examined both experimentally and computational (by our group and by others) through a study of the rat and human IAPP sequences. The study of IAPP is particularly challenging because the peptide is intrinsically disordered, in other words, it does not populate a single well-defined three-dimensional structure, but rather interconverts between a number of co-existing conformations. While not readily amenable to traditional ensemble-averaging experimentally (for instance CD studies [50]–[53] simply report that both human and rat IAPP variants are globally disordered and mainly adopts random coiled structures), recent experimental advances in studying IDPs have revealed subtle differences between IAPP variants. For instance, NMR studies looking at secondary chemical shifts [54]–[60] showed that IAPP variants are partially disordered but that the N-terminal part adopts helical structure, and that this structuring is significantly more pronounced in rIAPP than in hIAPP. A study combining data from CD, fluorescence dye binding, atomic force microscopy/AFM and electron microscopy/EM [61] showed that hIAPP adopts two distinct conformers containing both β-sheet and α-helix structural motifs. More recently, ion-mobility mass spectrometry studies [62], [63] and 2D-IR spectroscopy analysis [64], [65] showed evidence of the presence of β-structure in hIAPP monomeric and oligomeric samples. We recently explored the conformational space sampled by hIAPP and rIAPP using replica exchange molecular dynamics simulations (REMD) with an Amber force field ff96 and an implicit solvent (igb5). Our simulations showed that both peptides were flexible, and populated over 10 structural families (see supporting material of ref. [62]) even when using a very large dissimilarity measure (i.e. Cα-RMSD cutoff of 3 Å). Despite this flexibility, we were able to identify some very interesting structural similarities and differences between these two sequences. Whereas rIAPP was found to populate only helix-coil conformations, hIAPP populated both helix-coil conformations and β-rich conformations including a helix-hairpin and an extended β-hairpin. The structures obtained from simulation were a good match for the collision cross-sections obtained experimentally using ion-mobility mass spectrometry [62], [63]. Using GBSA implicit solvent model and Amber99SB force field, Murphy et al identified partially structured conformational states of the hIAPP monomer [66]. Using an explicit solvent and the Gromos96 53a6 force field, Reddy et al. independently obtained similar structures for both rIAPP and hIAPP, consistent with their 2D-IR data [64], [65]. Using the modified TIP3P water model and the CHARMM27 force field, Liang et al. [67] probed sequence-induced differences in structural stability between hIAPP and rIAPP from preformed monomer to pentamer, which is based on strand-loop-strand scaffold. Their simulations showed rIAPP adopt less β-sheet-rich structure and a disturbed U-shaped topology than hIAPP.

In the present paper, we perform an extensive investigation of the conformational space of two additional IAPP variants, the non-amyloidogenic pig variant (pIAPP) and the amyloidogenic cat variant (cIAPP). Additionally, we extend our simulations of the rat and human forms to match the simulations lengths (600 ns/replica) that we use in this study. To our knowledge, there are no published studies of the pig and cat IAPP structures. By enlarging our dataset of IAPP variants and using available peptide-activity data, we are now able to formulate a novel structure-activity relationship that rationalizes the dual function (pathological and physiological) of IAPP.

## Results

Details of the methodology are given in the Methods section. We used the replica exchange molecular dynamics protocol (REMD), with the Amber ff96 force field coupled with the implicit igb5 solvent model to sample to conformational space of IAPP. REMD simulations were performed for each IAPP variant (pIAPP, rIAPP, cIAPP and hIAPP), initiated from an extended conformation and run for 600.0 ns per replica, leading to 9.6 µs for each variant (16 replicas per variant). Block analysis was used to ensure convergence (see Table S2 in Text S1), and analysis was performed on the last three blocks of the trajectory (100.0 ns per block) at temperature of 300 K.

### The non-amyloidogenic rat and pig sequences have higher helical and lower sheet content than their amyloidogenic human and cat counterparts

**Figure 3:**
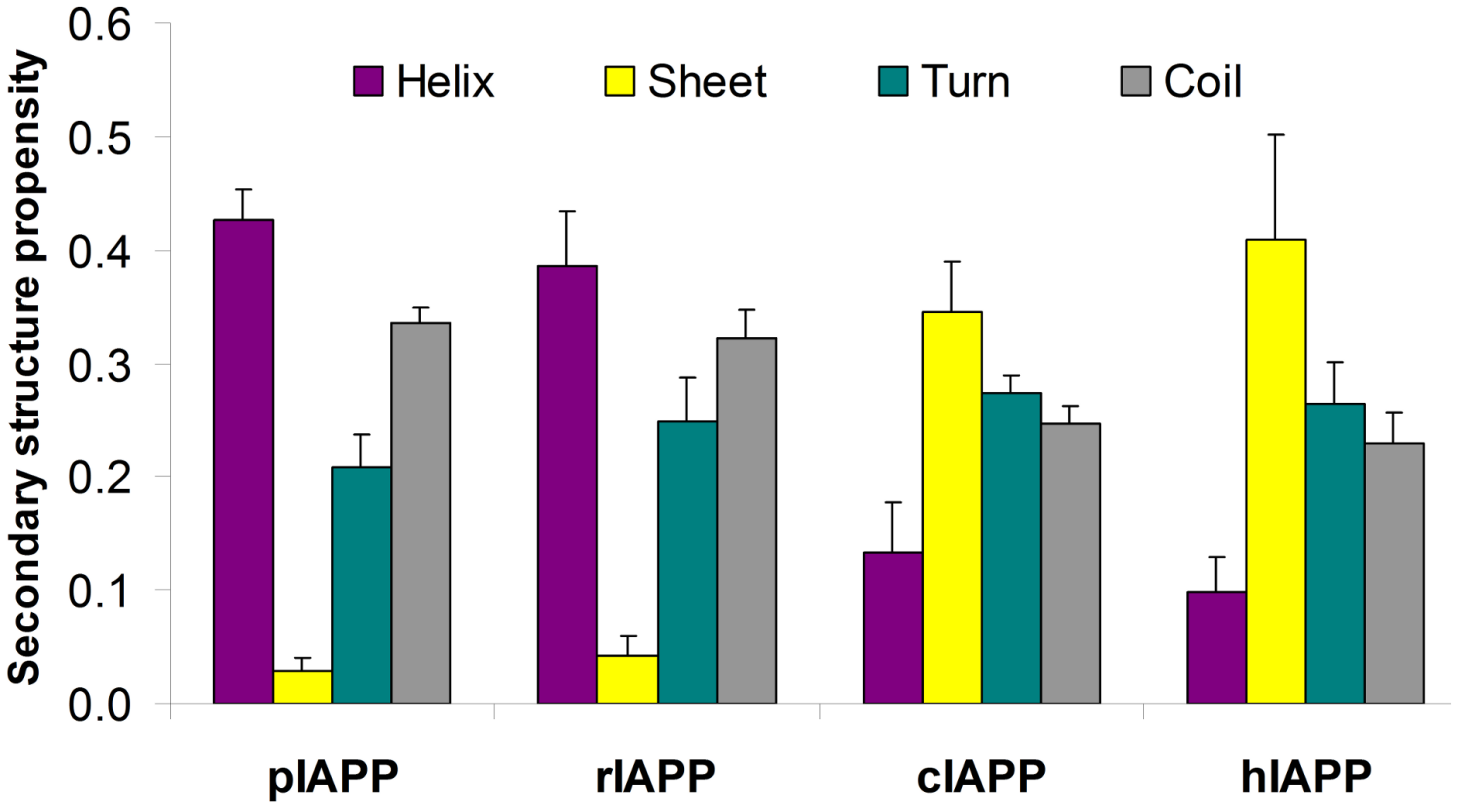
Secondary structure propensities of the four IAPP variants at 300 K. Stardard deviation was calculated from the last 3 blocks of the simulation data (100 ns of each) at 300 K.

The secondary structure propensities for the four peptides are shown in Figure 3. While all peptides show a high fraction of turn and coil structures (from 0.49 for hIAPP to 0.57 for rIAPP), consistent with the natively disordered structural nature of IDPs, there are nonetheless some striking differences between the amyloidogenic (human, cat) and non-amyloidogenic (rat, pig) sequences. In particular, while helicity is present in each peptide, the degree of helicity is much more pronounced for the non-amyloidogenic sequences (∼0.4 for pIAPP and rIAPP vs. ∼0.1 for cIAPP and hIAPP ). The trend for sheet structure is reversed, with low β-sheet content (∼0.03) for pIAPP and rIAPP and significantly larger content (∼0.4) for cIAPP and hIAPP.

**Figure 4:**
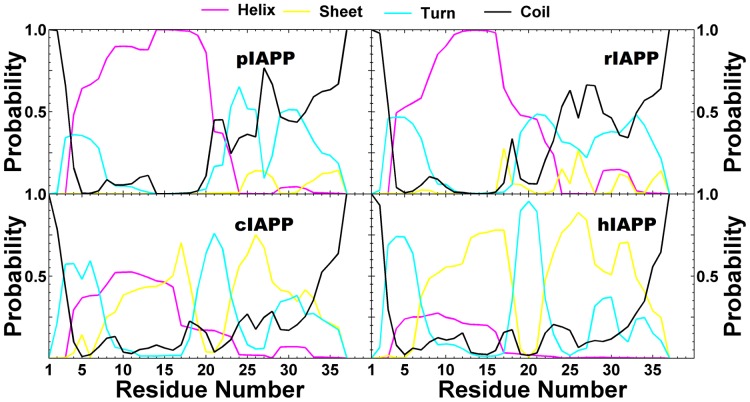
Position-dependent secondary structure propensities for the four IAPP variants from the last 300 ns at 300 K.

The location of the secondary structure elements on a per-residue level is shown in Figure 4. Overall, the two non-amyloidogenic sequences (pIAPP and rIAPP) share similar secondary structural profiles, while the amyloidogenic sequences (cIAPP and hIAPP) share a different pattern. For the non-amyloidogenic sequences, conserved region I (residues 1–16) consists primarily of helical structure, whereas the mutation region (residues 18–29) and conserved region II (residues 30–37) consist primarily of turns and coils, with a modest amount of helix and sheet structure. For the amyloidogenic sequences, the conserved region I (residues 1–16) shows both helical and sheet elements, with the sheet contribution much more pronounced. The mutation region (residues 18–29) now show a turn at residues 18–22, linking the N-terminal (residues 5–18) strand to a C-terminal (residues 22–33) strand (the latter located in conserved region II (residues 30–37)), indicative of the presence of β-hairpin population.

### The non-amyloidogenic sequences populate helix-coil and helix-hairpin structures, while the amyloidogenic sequences adopt an additional β-hairpin structure

**Figure 5:**
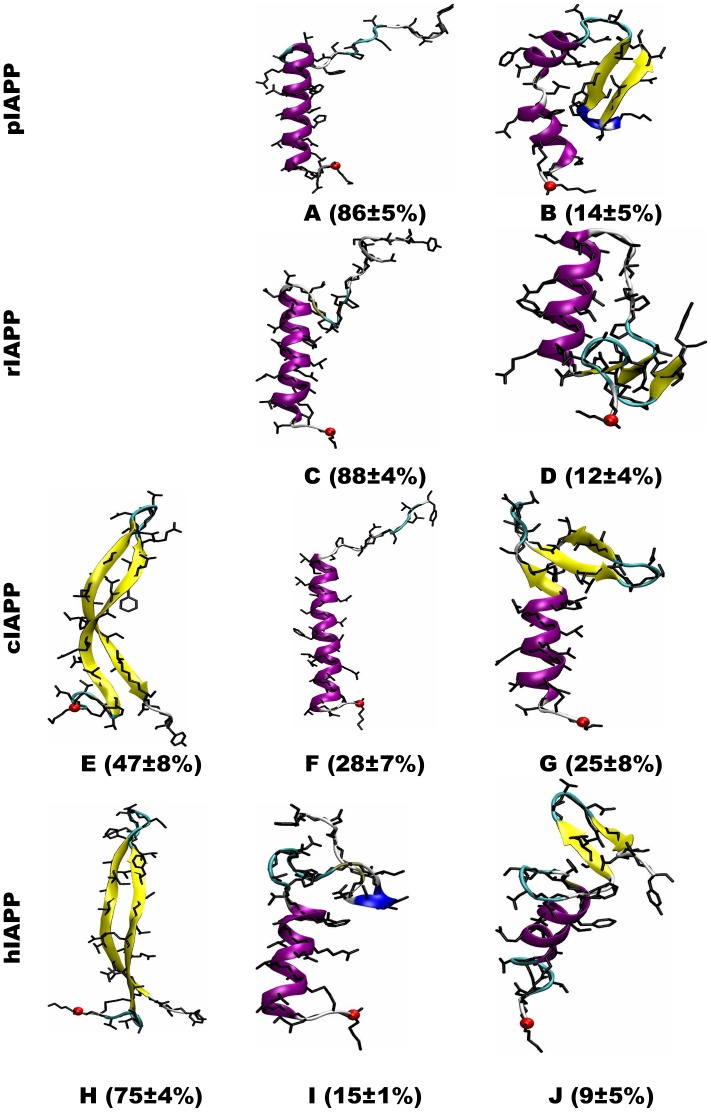
Representative structures of super structural families for each IAPP variant. Standard deviation was calculated from the last 3 blocks of the simulation data (100 ns of each) at 300 K (See Figures S2 for all structural families in each super family from the last block). The backbone is shown in cartoon and the secondary structure is coded by color: coil in silver, β-sheet in yellow, isolated β-bridge in tan and turn in cyan. The N-terminus is indicated by a red ball.

From our clustering analysis (described in the Methods section), a large number of diverse structural families were identified. The centroid structures of the top 15 most populated structural families (≥1% of total structure population) from the last 100.0 ns of simulation are shown in Table S3 of Text S1 for each IAPP sequence. The structural families were then further merged into several super structural families based on similarity in the molecular topology. A representative structure and the abundance for each super structural family are presented in Figure 5.

The non-amyloidogenic pIAPP and rIAPP structural ensembles contain two super families: a helix-coil super family (structures **A** and **C** in Figure 5) and a helix-hairpin super family (structures **B** and **D** in Figure 5). In the helix-coil fold, the peptide adopts a short turn-coil (residues 1–7), a short helix (residues 8–17) and a long turn-coil (residues 18–37). In the helix-hairpin fold, the peptide adopts a short turn-coil (residues 1–7), a short helix with a kink (residues 8–17) and a short β-hairpin close to the N-terminal (β-strands: residues 25–27 and 33–36, loop: residues 28–31). The helix-coil supper family (∼87%) is more abundant than the helix-hairpin super family (∼13%). Although pIAPP differs from rIAPP by 9 out of 37 residues, their tertiary structure ensembles are very similar (as are their secondary structural profiles, as seen in Figure 4).

The cIAPP and hIAPP structural ensembles contain three super families: a β-hairpin super family (structures **E** and **H** in Figure 5) (a β-strand 9–17, turn 18–22, and another β-strand 23–33), a helix-coil super family (structures **F** and **I** in Figure 5), and a helix-hairpin super family (structures **G** and **J** in Figure 5). The first two super families are very similar to the two super families adopted by pIAPP and rIAPP, but occur in different abundance. The difference is particularly striking in the case of the helix-coil super family, with a population of ∼28% for cIAPP and ∼15% for hIAPP, dramatically less than the ∼87% seen for pIAPP and rIAPP. The helix-hairpin super family population is modest for all sequences (∼25% for cIAPP, ∼9% for hIAPP, and ∼13% for pIAPP and rIAPP). The β-hairpin fold is only seen for the amyloidogenic sequences, and occurs with large population (∼47% for cIAPP and ∼75% for hIAPP).

Although the cIAPP structural ensemble is quantitatively similar to that of hIAPP, we identified a number of quantitative differences. In particular, the population of the hairpin super family is ∼28% less for cIAPP than for hIAPP, and the population of helix-coil super family of cIAPP is correspondingly larger (by ∼13%). Subtle differences (for instance, a shift or difference in the strand length) are observed (see Table S3 of Text S1 in which the top 15 structural families are shown).

### The β-hairpin is less soluble than the helix-coil structure

Peptide solubility is an important component in the aggregation process. We computed the GBSA solvation energy for each of the IAPP super families (Table 1). The absolute GBSA solvation energies of these ionic peptides are large (<−554 kcal/mol) as a result of the charges (+4 of pIAPP and +3 of rIAPP, cIAPP and hIAPP) carried by the four peptides. Based on the order of the GBSA solvation energy, we find that the β-hairpin is the least soluble motif, the helix-coil the most soluble with the helix-hairpin lying in the middle. When the relative GBSA of the four IAPP variants is considered (using hIAPP as the zero scale reference), the order of solubility is rIAPP (−97.1 kcal/mol)>pIAPP(−90.7)>cIAPP (−74.3)>hIAPP (0.0 kcal/mol). This order correlates with the aggregation ability order of the four IAPP variants, with the non-amyloidogenic sequences being more soluble than the amyloidogenic ones.

**Table 1:** Solvation energy of the super structural families of the four IAPP variants (Fig. 5).

	Charge	Helix-coil	Helix-hairpin	β-hairpin	Average	Relative^2^
		GBSA(kcal/mol)	GBSA(kcal/mol)	GBSA(kcal/mol)	GBSA(kcal/mol)	Δ(kcal/mol)
**pIAPP**	+4	−648.0±30.3	−620.6±30.0	-	−645.3±27.3	−90.7
**Δ** 1		0	27.4	-		
**rIAPP**	+3	−652.5±25.9	−643.7±20.4	-	−651.7±25.2	−97.1
**Δ** 1		0	8.8	-		
**cIAPP**	+3	−657.2±24.6	−638.5±21.5	−610.8±22.6	−628.9±29.8	−74.3
**Δ** 1		0	18.7	46.4		
**hIAPP**	+3	−585.3±22.9	−567.8±21.0	−547.0±15.9	−554.6±23.3	0
**Δ** 1		0	17.5	38.3		

Standard deviation was calculated from the last 3 blocks of the simulation data (100 ns of each) at 300 K. GBSA (Solvation energy): Generalized Born electrostatic solvation energy + non-electrostatic surface solvation energy term.

1 relative to helix-coil;

2 relative to hIAPP.

### Dynamic fluctuation (RMSF) at residue-level

**Figure 6:**
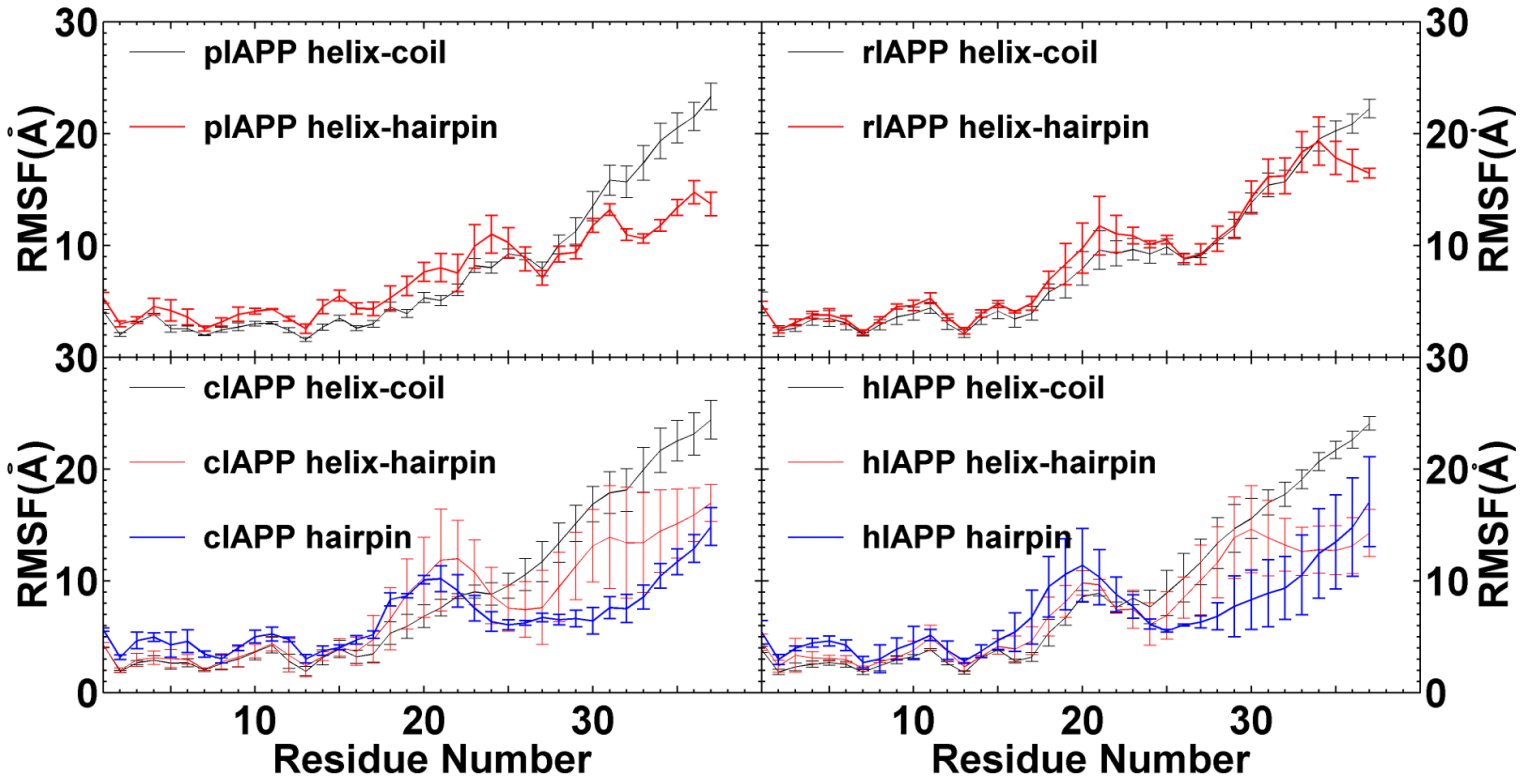
Root mean square fluctuation (RMSF) of each residue (Cα atom) of the four IAPP variants in the super structural families at 300 K. Residues 1–17 were aligned preceding the RMSF calculations.

Since IAPP is an IDP, and, as such, populates partially structured conformers, it is important to consider the structural flexibility of the conformations identified in simulation. Our structural ensembles enable us to directly characterize this feature for the folds of the four IAPP variants by calculating their RMSF (see Methods section). These results are reported in Figure 6. The helix-coil fold of all four IAPP variants show much smaller structural fluctuation in the N-terminal part (residues 1–17, where the helix is located) (RMSF of ∼5 Å) than in of the C-terminal part (residues 18–37), with an approximately linear increase from ∼5 Å to ∼20 Å. The flexibility of the N-terminal region may be required for the hormone function of IAPP (i.e. interacting multiple membrane receptors). In the case of the helix-hairpin fold of the four IAPP variants, the N-terminal part (residues 1–22) has comparable fluctuations to the same region in the helix-rich fold, but the C-terminal part (residues 30–37) is slightly more rigid than the corresponding part in the helix-coil fold (by ∼2 Å). In the case of the β-hairpin fold seen only in the amyloidogenic cIAPP and hIAPP sequences, the N-terminal part (residues 1–22) has comparable fluctuations to the same region of their helix-coil fold, but the C-terminal part (residues 23–37) is significant more rigid than the corresponding part of the helix-coil fold (by ∼5 Å).

## Discussion

All IAPP sequences play the same physiological role in reducing post-meal blood glucose [2], however, some sequences are capable of aggregating into pathological structures. In this paper, we used all-atom REMD simulations coupled with an implicit solvent model to thoroughly sample the conformations adopted by two amyloidogenic sequences of IAPP (cat and human) and two non-amyloidogenic sequences (rat and pig). We wished to examine whether structural similarities existed between non-amyloidogenic and amyloidogenic forms of IAPP that could explain the dual functional/pathological roles that certain IAPP variants play. The similarity in functions suggests a possible similarity in structure. On the other hand, the fact that a few point mutations lead to enhanced aggregation tendencies suggests that these mutations may lead to dramatic conformational changes at the monomeric level, shifting the population from functional to pathological.

Our simulations revealed that all four peptides populated helix-coil and helix-hairpin conformations, but that the amyloidogenic sequences populated in addition a β-hairpin conformation. The helix-coil structure was the dominant fold for the non-amyloidogenic structures, and the second dominant fold for the amyloidogenic sequences (after the hairpin). We propose that this fold corresponds to the physiologically relevant fold. The helical region in this fold is located in the N-terminal region, a region conserved in all CT peptides. NMR studies on rat and human variants support the presence of helicity in the N-terminal region [54], [56], [59], [68]. We found that this helical region was the most rigid region of all folds. Intriguingly, the C-terminal (turn/coil) region, corresponding to conserved region 2, is the most flexible part. Both conserved regions 1 and 2 are involved in receptor binding, and it is possible that this dual rigid/flexible architecture may play a role in enabling IAPP to bind to multiple AMY_1–3_ receptors [7], [22]. Given the lower abundance (∼22%) of the helix-coil conformation adopted by amyloidgenic peptides (cIAPP and hIAPP) relative to that (∼87%) of non-amyloidogenic peptides (pIAPP and rIAPP), we would expect hIAPP and cIAPP to have slightly reduced normal hormone function relative to pIAPP and cIAPP. Indeed, Young et al.[2] have shown in a preclinical rat study that hIAPP has slightly lower binding affinities for amylin, CGRP, and calcitonin receptors, and induces slightly weaker responses in isolated muscle (Table 2).

**Table 2:** Diminished amount of putative helix-coil hormone-competent conformation in hIAPP relative to rIAPP seen in simulation, and its link to diminished normal hormone function of hIAPP relative to rIAPP.

	Helix-coil	Amylin receptors	CGRP receptors	Calcitonin receptors	Inhibition of gastric	Responses in isolated
	abundance^1^	K_i_ (nM)^2^	K_i_ (nM)^2^	K_i_ (nM)^2^	emptying ED_50_ (ug)^2^	muscle EC_50_ (nM)^2^
rIAPP	88±4%	0.024±0.002	3.8±1.1	2.7±1.5	0.18±0.10 log	4.97±0.10 log
hIAPP	15±1%	0.030±0.006	9.3±2.5	10.1±0.7	0.13±0.14 log	7.63±0.06 log

1 from this study;

2 Binding and biological activity data from reference 2.

In contrast, the β-hairpin structure, present only in the amyloidogenic sequences, is a possible candidate for an amyloid-competent structure. β-hairpin structures have been found in molecular dynamics simulations in a number of amyloidogenic peptides, most notably in fragments of the Alzheimer Amyloid Aβ peptide [69]–[83] and the prion protein [84], [85].Simulations of oligomerization of the Aβ(25–35) peptide indicate that hairpins play an important role in initiating aggregation and in stabilizing the growing front of the fibril [83]. This may also be the case for IAPP. The hairpin structure that we find in simulation shares important similarities with the structure of hIAPP in the context of a fibril. The solid state NMR [86] structure of the hIAPP fibril consists of a strand-loop-strand topology (related to the strand-turn-strand hairpin by a 90° rotation), with the loop (residue 18–27) located at the turn region of our hairpin. By having the correct strand placement (as in the fibril), the hairpin structure could facilitate nucleation and subsequent fibril growth. Further support for the notion of this structure as a key player in aggregation comes from the work of Kapurniotu and co-workers who identified through fragment binding affinity studies a number of “hot-spot” regions responsible for inter-peptide interactions in aggregation [87]. These hot-spots correlate with the β-strand regions of the hairpin seen in our simulations [62].

The β-hairpin structure is less soluble and less flexible than the helix-coil fold based on our analysis of the GBSA solvation energy and Cα-RMSF, features that make it a good candidate for β-sheet formation. Indeed, this increased rigidity might contribute to the fast association of β-hairpins into β-sheet rich oligomers. Our recent dimer simulations of hIAPP and rIAPP [63] support this picture: the β-rich monomers have strong tendencies to form β-rich dimers, while the helix-coil rich monomers form, if anything, only loosely bound, disordered complexes with much lower binding energies. Formation of β-rich hIAPP dimers was also found in atomistic, explicit solvent simulations [65] and both hIAPP dimers with moderate β-content and rIAPP dimers with no β-content were observed in a Hamiltonian-Temperature-REMD simulations using a coarse grained protein force field (OPEP) [88].

The notion of a hairpin as an important player in the aggregation process can explain a number of experimental observations. For instance, within this framework, the increased aggregation rates of the S20G IAPP mutation [25], [26] can be explained by an increased propensity to form a β-turn (glycines are turn promoters and residue 20 that is involved in this mutation lies right in the turn region of our hairpin (residues 18–22)). In addition, the observed inhibition effects of a non-aggregating form of hIAPP with two N-methylations at positions G24 and I26 (hIAPP-GI) could be explained by blocking inter-strand β-sheet formation [43], [89], [90]. In other words, the β-strand of hIAPP-GI could bind to the β-strand of hIAPP, forming a complex that now has a face with exposed N-methyl groups that is unable to hydrogen bond with another hIAPP peptide, thus blocking further growth.

cIAPP is less amyloidogenic than hIAPP [15] and, consistent with our hypothesis of a role of the hairpin in facilitating aggregation, our structural data shows that cIAPP has slightly lower β-sheet content (β-hairpin) than hIAPP. We speculate that the decrease in hairpin population is due to the S29P mutation that differentiates cat from human. Proline is indeed known to be a β-sheet breaker. Along similar lines, other disorder-promoting substitutions (e.g. P, R and K) [91] may further lead to a diminished propensity for hairpin formation, eventually leading, in the case of rIAPP (with key mutations A25P, S28P and S29P) and pIAPP (S20R and N31K) to the complete disappearance of the β-hairpin population. It is interesting to note that the drug PRAMLINTIDE (symlin) with same sequence as hIAPP, but containing the 3 proline substitutions of rIAPP, shows a very weak tendency to aggregate [58]and has proven to be an efficacious agent that takes over the physiological role of hIAPP, acting as a synergistic partner to insulin [2], [92].

Interestingly, we find that all four sequences adopt a helix-hairpin fold, although in lower amounts than the helix-coil fold (for pIAPP and rIAPP) and hairpin (for hIAPP and cIAPP). This fold is more soluble than the β-hairpin fold, but less soluble that the helix-coil fold. We speculate that this motif may act as an on-pathway intermediate leading to β-rich conformations (β-hairpin or β-sheet) and thus amyloid fibrils under certain conditions (e.g. the presence of an interface, peptide-peptide interaction, solvent effect etc.). Indeed, rIAPP, although commonly thought of as a non-aggregating species, has been observed to aggregate into fibrils under specific non-physiological conditions [93]–[95]. Indeed β-sheet-rich fibrils of rIAPP were seen to form at a liquid-solid interface [93], mixing rIAPP with hIAPP lead to a templating of rIAPP onto hIAPP fibrils [94], and placing rIAPP in Tris-HCl buffer and sonicating lead to fibril formation [95].

We note that it is also plausible, as proposed by Miranker and coworkers [54], Eisenberg and coworkers [57] and Raleigh and coworkers [96], that early oligomerization may be initiated by helix-helix association, with β-structure emerging later in the aggregation process. It is of course difficult to tell whether experimentally observed helix-rich oligomers are on or off-pathway to fibrils. Likewise, the hairpins that we see in simulation may be on-route to fibrils formation, or may as well lead to off-pathway aggregates. However, small hairpin oligomers, even if not directly on-route to fibril formation, may play an important role in toxicity.

Small oligomers are also increasingly being associated with hIAPP cytotoxicity [35], [37]. In particular, membrane pores, formed by small amyloidogenic oligomers, have been suggested as a means of toxicity of hIAPP [97]. These pores can be formed by helical conformers of hIAPP, as supported by experiments on the 1–19 fragment of IAPP. Indeed, the human and rat IAPP(1–19) fragments can adopt helical conformation in membrane mimics [56] and have been shown to be toxic to cells, with hIAPP(1–19) being more toxic than rIAPP (the latter differ by an H to R substitution) [98]. We note that it is possible that IAPP pores can also be formed from β-rich conformation, as in the case of the β-rich annular-like channel proposed for Aβ and other amyloid peptides [99], [100], based on molecular dynamics simulations, atomic force microscopy and channel conductance measurements. A similar β-rich annular-like channel model for hIAPP has recently been proposed using molecular dynamics simulations [101]. The hairpin structures that we observe are reminiscent of the cylindrin structures discovered by Eisenberg and co-workers, cylindrical barrels of β-hairpins that may interact with membranes and constitute a generic architecture for toxic amyloid oligomers [102]. There is at present no experimental data for a cylindrical barrel model for hIAPP, but such a structure is plausible given the observed β-hairpin in simulations and the observation of β-barrel type of ion channel for other amyloid systems. Finally, another toxicity mechanism of hIAPP may be associated with membrane fragmentation due to the growth of amyloid fibrils [103].

**Figure 7:**
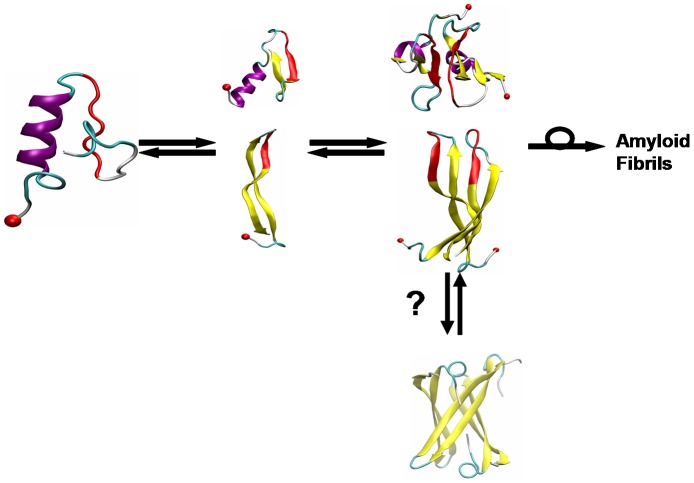
Schematic representation of misfolding/aggregation mechanism of hIAPP. Left: Helix-coil structure for normal function; Middle: aggregation-prone β-rich monomers; Right: early putative toxic dimers (Dupuis *et al. JACS 2011*). The question mark indicates the hairpins may further form cylindrin-like toxic oligomers, modeled from the cylindrical barrel of an amyloid peptide (PDB id: 3SGR) (Laganowsky et al. Science 2012). The isomerization symbol indicates that at some, as yet unknown size, the β-strand aggregates must rearrange to the β-sheet aggregates found in the fibrils. N-terminus is indicated by red ball, residues 23–29 in the “mutation region” are in red. In the β-hairpin, residues 19S-20S-21N-22N form a turn and residues 11–19 and 23–33 form two β-strands.

In summary, there are compelling genetic, biochemical, cellular and animal data to support both natural biological functions for hIAPP and a toxic role of hIAPP leading to β-cell death [14], [34], [37], [104]–[107]. Combining these functional data with our structural models of four IAPP variants, we put forth the structure-activity relationship (SAR) that **the helix-coil conformations are responsible for the normal hormone function of IAPP; and that β-rich conformations of IAPP may be linked to β-rich aggregation and contribute, along with other mechanisms, to the toxicity of IAPP.** While the former might be realized by binding of the helix-coil conformers to AMY receptor, the latter might be due to the formation of toxic β-rich oligomers and amyloid fibrils leading to β-cell death. This SAR scheme is summarized in Figure 7. Our SAR can give insights into the rational design of drugs to combat Type II Diabetes, with drugs that either destabilize the pathological conformations and/or promote the formation of the physiologically active conformations. These drugs could come in the form of small molecules, or be peptide based. Ideally, one could design an IAPP variant that retains the functional role of hIAPP, but does not have the same tendency as hIAPP to misfold and aggregate (such as pramlintide), and that furthermore inhibits the aggregation of wild type hIAPP. This drug would not only enhance insulin-sensitivity (like pramlintide), but also preserve β-cells by preventing aggregation.

## Methods

The AMBER 8 [108] simulation suite was used in replica exchange molecular dynamics (REMD) [109] simulations. The four IAPP variants were modeled using the AMBER all-atom point-charge protein force field, ff96 [110]. Solvation effects were represented by the implicit solvent model (IGB = 5) (96) plus the surface term (gbsa = 1, 0.005 kcal/Å2/mol) with an effective salt concentration of 0.2 M. Studies in the Dill group have examined a number of force field/implicit solvent combinations [111], [112] and have concluded that this ff96/IGB5 offers a good balance between helical and sheet propensities. This combination, in conjunction with REMD simulation yielded impressive results in the folding of both small α, β and α/β proteins with a well defined native fold [111], [113]–[115] and natively unfolded peptides [62], [84] including hIAPP and rIAPP, and in predicting correct inter-domain orientation of a large multi-domain protein (CheA) [116].

The simulation protocol closely followed the one described in the Methods section of reference [62] and the salient points are highlighted here. A minimized extended-conformation of each IAPP variant was used as the input for each set of REMD simulations. 16 replicas were set up with initial temperatures exponentially spaced from 270 to 465 K, which were optimized by the algorithm described in reference [117]. Initial atom velocities for each replica system were generated according to the Maxwell-Boltzmann distribution corresponding to the initial temperature of that replica. The first 1.0 ns of molecular dynamics simulation was performed without replica exchanges to equilibrate the system at its target temperature. After the equilibrium phase, exchanges between neighboring replicas were attempted every 2000 MD steps (3.0 ps) and the exchange rate was ∼20% in the production phase. SHAKE [118] was applied to constrain all bonds linking to hydrogen atoms and a shorter time step of 1.5 fs rather than the typical 2.0 fs was used to circumvent the occasional SHAKE failure, probably caused by large atomic displacements at the high temperatures used in our simulations (up to 450 K leading to high kinetic velocities). In order to reduce computation time, non-bonded forces were calculated using a two-stage RESPA (reference system propagator algorithm approach) [119] where the fast varying forces within a 12 Å radius were frequently updated (e.g. every step) and those beyond 12 Å were updated every two steps. Langevin dynamics was used to control the target temperature using a collision frequency of 1.0 ps^−1^ (a low collision frequency is used for better conformational sampling). The center of mass translation and rotation were removed every 500 MD steps (0.5 ps). Each replica was run for 600.0 ns giving a cumulative simulation time of 9.6 µs for each IAPP system The snapshots in the replica trajectories were saved at 45.0 ps intervals for further analysis.

### Convergence of the REMD simulations

Because experiments are typically performed around 300 K, our data analysis was focuses on the replica at 300 K. The convergence was rigorously checked by a block analysis: the total 600.0 ns sampling at 300 K was equally divided into six blocks, and structural properties was calculated for each block. For the four sets of REMD simulations, a good convergence was found during the last half of the trajectory (see for example, the secondary and tertiary structure data of the four IAPP variants in Table S2 of Text S1). Thus, the standard deviations of the structural properties presented in the main text were calculated from the last three blocks (i.e. the last 300.0 ns).

### Secondary structure assignment and tertiary structure clustering

The STRIDE program of Frishman and Argos [120] was used to obtain secondary structure propensities. For tertiary structure analysis, the structural ensembles from simulations were classified into structural families using the GROMACS clustering protocol [121], in which the structure similarity metric is based on a pair wise Cα- RMSD (root mean square deviation) cutoff of 3.0 Å, the neighboring structures are identified for every structure using the RMSD similarity cutoff, the structure having the most neighbors (called as the centroid structure) is removed together with its neighbors to form a structure family, and the process continued for the remaining structures until all structures have been assigned into the structural families. The centroid structure serves as the representative structure of the structural family. For example, the centroid structures of the top 15 populated structural families (≥1% of total structure population) from the last 100.0 ns for each IAPP sequence are shown in Figure S3. Next, the structural families were further merged into three super-families (helix-coil, helix-hairpin and β-hairpin) based on the secondary structure type of both the N-terminal part (residues 1–17) and the C-terminal part (residues 18–37): First, it belongs to a helix-rich fold if the N-terminal part contains more helix than β-sheet, otherwise it belongs to β-hairpin super family; Second, a helix-rich fold belongs to the helix-hairpin super family if the C-terminal part contains more than four sheet-residues coil (i.e. a minimal 2∶2 β-hairpin), otherwise it belongs to the helix-coil super family.

### Dynamic fluctuations

Dynamic fluctuations of each residue was characterized by calculating the Root Mean Square Fluctuation (RMSF) of its Cα atom from the structural ensemble. Because the N-terminal part (residues 1–17) of these IAPP peptides is more rigid than its C-terminal part (residues 18–37), a superposition of the C-terminal part was carried out prior to the RMSF calculation. Also because the four IAPP variant contains two or three types of folds (helix-coil, helix-hairpin and β-hairpin), the RMSF was calculated separately for each one.

### GBSA solvation energy

The absolute solubility of the four IAPP variants can be estimated from their solvation free energies. When the solute conformational entropies of the IAPP variants are comparable, the relative solvation free energy can be estimated from the relative GBSA solvation energy. A recent benchmark study [122] has shown that GBSA models give reliable results when only the relative solvation energy is considered. We obtained the statistics of GBSA solvation energy for each IAPP fold from its last 300.0 ns of simulation data at 300 K.

## Supporting Information

Text S1 Supplemental data showing 1) the calcitonin family of peptides, 2) convergence of simulations and 3) the main structural families for each IAPP peptide based on a clustering analysis.
(PDF)
