# Supplementary material for: Structural Similarities and Differences between Amyloidogenic and Non-Amyloidogenic Islet Amyloid Polypeptide (IAPP) Sequences and Implications for the Dual Physiological and Pathological Activities of These Peptides
Source: PLoS Comput Biol. 2013 Aug 29;9(8):e1003211. doi: 10.1371/journal.pcbi.1003211 (PMC3757079; doi:10.1371/journal.pcbi.1003211)
Supplement: Text S1 — Supplemental data showing 1) the calcitonin family of peptides, 2) convergence of simulations and 3) the main structural families for each IAPP peptide based on a clustering analysis. (PDF) [file pcbi.1003211.s001.pdf]

Supporting material (Text S1) for:

Structural similarities and differences between amyloidogenic and non-amyloidogenic Islet Amyloid Polypeptide (IAPP) sequences and implications for the dual physiological and pathological activities of these peptides

Author Affiliation:

Chun Wu, Joan-Emma Shea [*]

Department of Chemistry and Biochemistry, University of California Santa Barbara,

Santa Barbara, CA 93106

Table S1. The calcitonin family of peptides. Three structural features are conserved (conserved residues are in red): 1) a disulfide bond close to N-termini; 2) a region of putative amphipathic α-helix; 3) a C-terminal amidation. CGRP: calcitonin gene-related peptide

	S–S
Human IAPP	KC NTATCATQRLANFLVHSSNNFGAILSSTNVGSNT Y-NH2
Calcitonin	CGNLSTCMLGTYTQDFNKFHTF PQTAIGVGA P-NH2
Adrenomedullin	GC RFGTCTVQKLAHQIYQFTDKDKDNVAPRNKISPQGY-NH2
αCGRP	AC DTATCVTHRLAGLLSRSGGVVKNNFVPTNVGSKA F-NH2
βCGRP	AC NTATCVTHRLAGLLSRSGGMVKSNFVPTNVGSKA F-NH2

Table S2 the convergence within last 300 ns of the REMD simulations. A total 600 ns sampling at 300 K is equally divided into six blocks for secondary and tertiary structure Hanalysis. A, E: pig IAPP; B, F: rat IAPP; C, G: cat IAPP; D, H: human IAPP

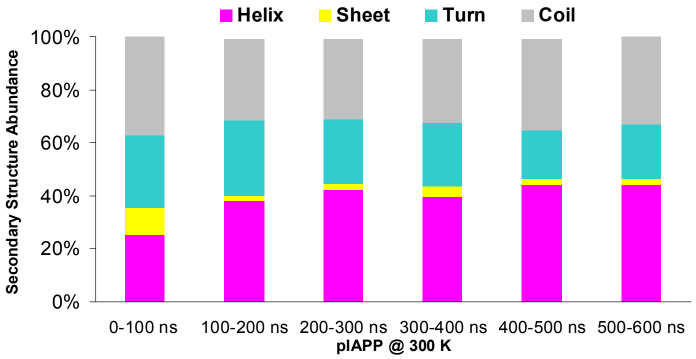

A

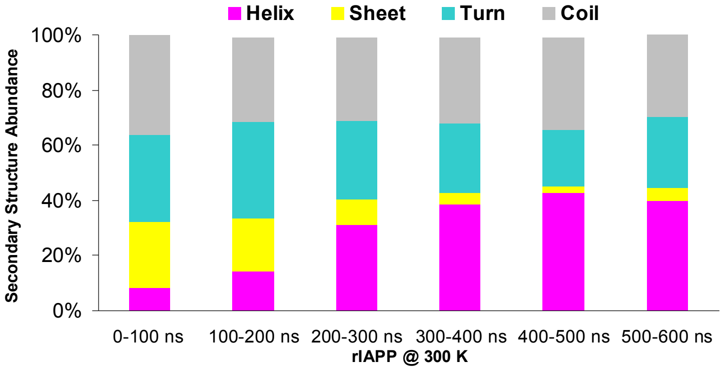

B

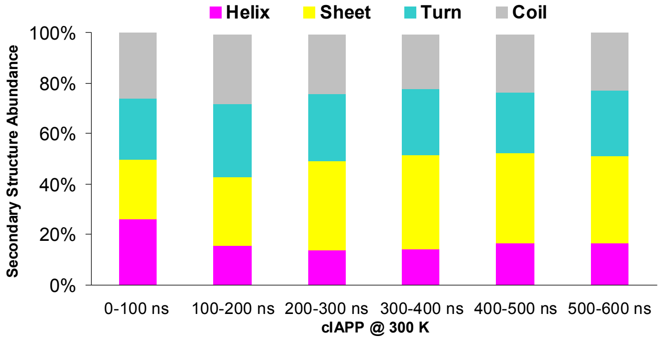

C

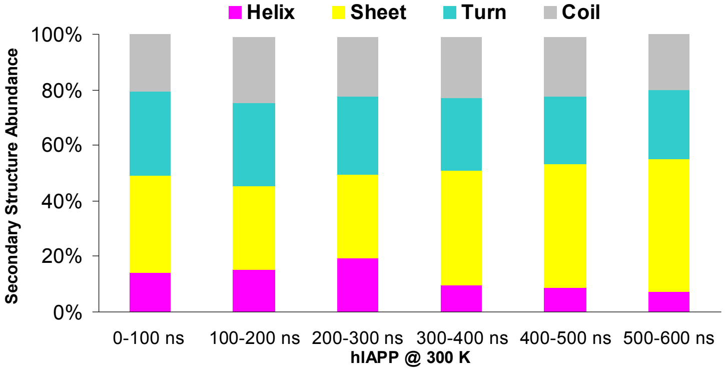

D

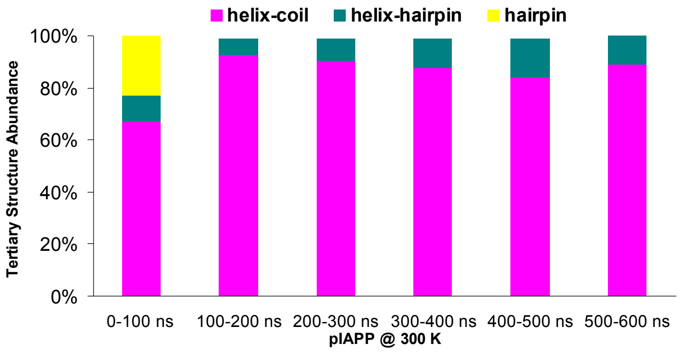
helix-coil
helix-hairpin
hairpin
100%
80%
60%
40%
20%
0%
Tertiary Structure Abundance
0-100 ns
100-200 ns
200-300 ns
300-400 ns
400-500 ns
500-600 ns
pIAPP @ 300 K

E

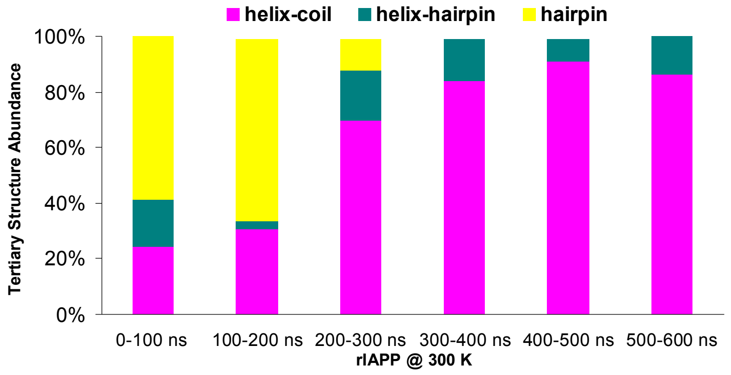
helix-coil
helix-hairpin
hairpin
100%
80%
60%
40%
20%
0%
Tertiary Structure Abundance
0-100 ns
100-200 ns
200-300 ns
300-400 ns
400-500 ns
500-600 ns
rIAPP @ 300 K

F

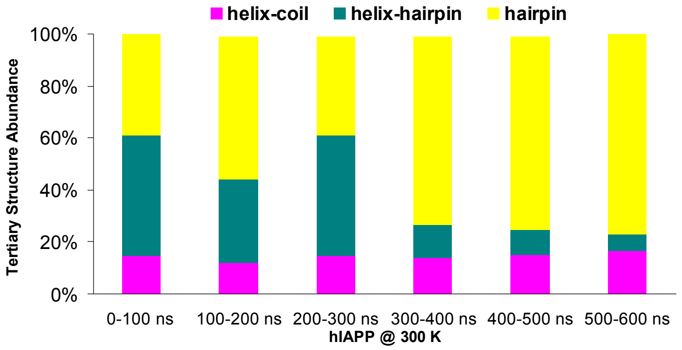
helix-coil
helix-hairpin
hairpin
100%
80%
60%
40%
20%
0%
Tertiary Structure Abundance
0-100 ns
100-200 ns
200-300 ns
300-400 ns
400-500 ns
500-600 ns
hIAPP @ 300 K

G

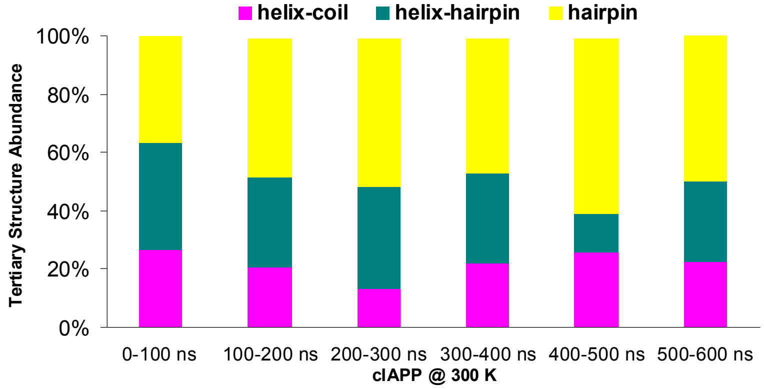
helix-coil
helix-hairpin
hairpin
100%
80%
60%
40%
20%
0%
Tertiary Structure Abundance
0-100 ns
100-200 ns
200-300 ns
300-400 ns
400-500 ns
500-600 ns
cIAPP @ 300 K

H

Table S3 Representative structures of the top 15 structural families for each IAPP peptide from the clustering analysis (500-600 ns) with a cutoff Cα rmsd of 3.0 Á. A1-B1: pig IAPP, C1-D1: rat IAPP, E1-G6: cat IAPP, H1-J6 human IAPP. The abundance is shown in parenthesis. The backbone is in cartoon; α-helical, β-sheet, β-bridged, turn and coiled conformations are colored in purple, yellow, tan, cyan and white respectively. The N-terminus is shown by a red ball.

Pig IAPP

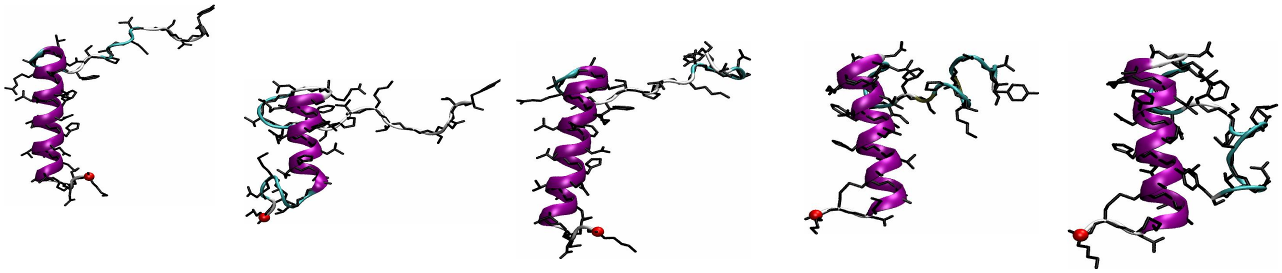

A1(11%) A2(4%) A3(3%) A4(3%) A5(3%)

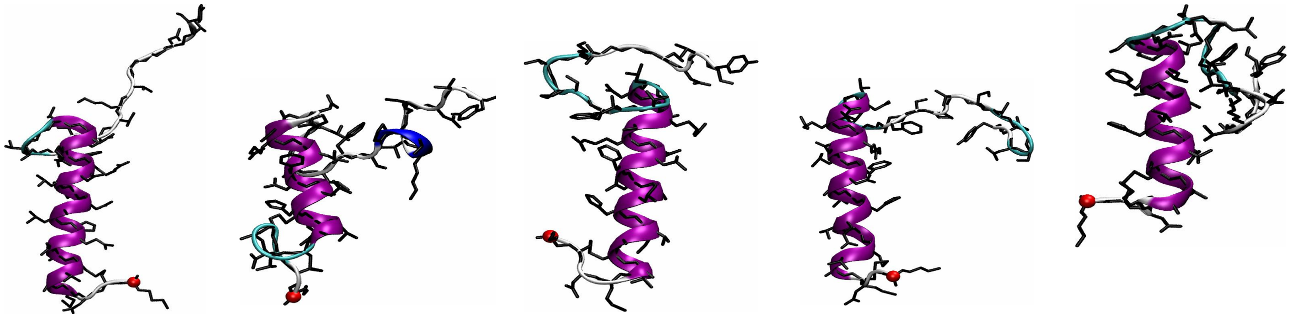

A6(3%) A7(3%) A8(2%) A9(2%) A10(2%)

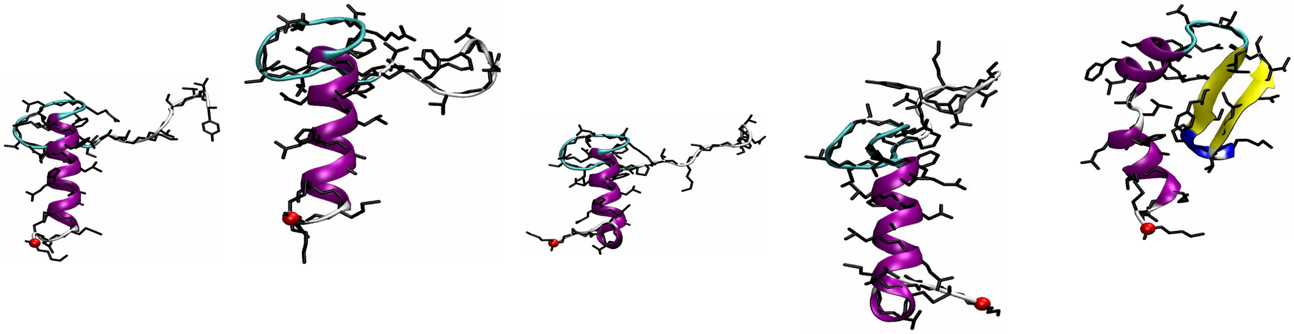

A11(2%) A12 (2%) A13(2%) A14(1%) B1(6%)

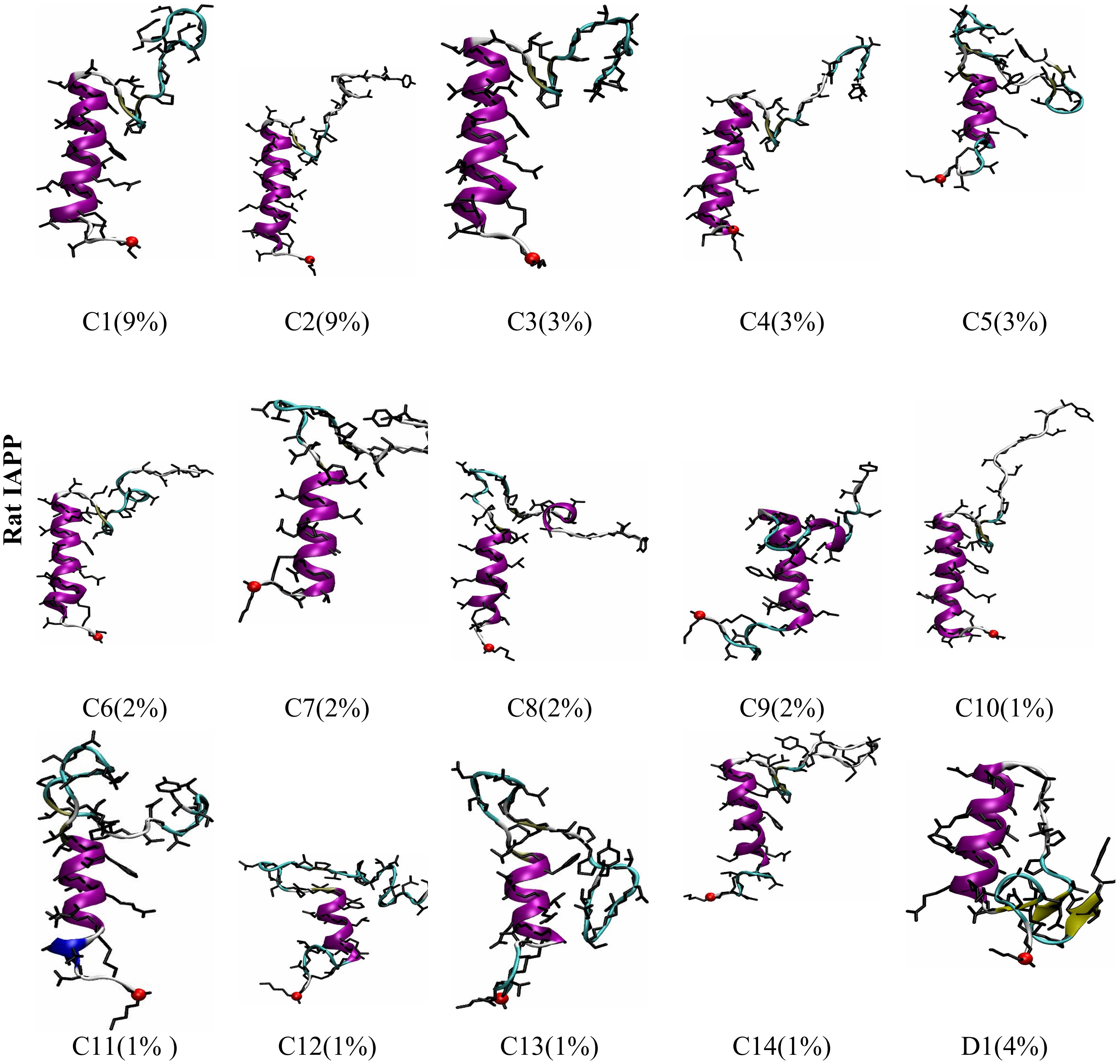
Rat IAPP
C1(9%)
C2(9%)
C3(3%)
C4(3%)
C5(3%)
C6(2%)
C7(2%)
C8(2%)
C9(2%)
C10(1%)
C11(1%)
C12(1%)
C13(1%)
C14(1%)
D1(4%)

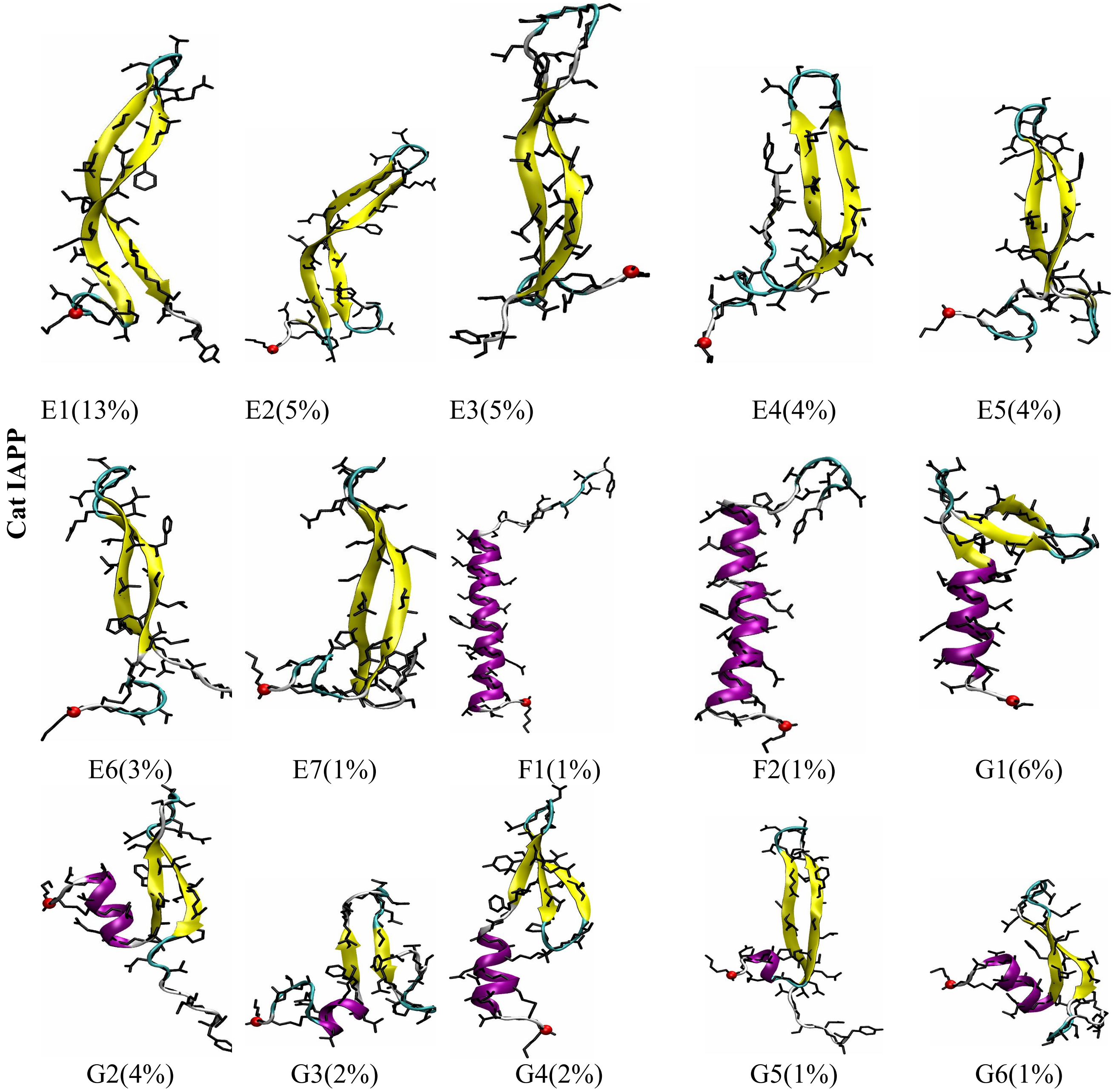
Cat IAPP
E1(13%)
E2(5%)
E3(5%)
E4(4%)
E5(4%)
E6(3%)
E7(1%)
F1(1%)
F2(1%)
G1(6%)
G2(4%)
G3(2%)
G4(2%)
G5(1%)
G6(1%)

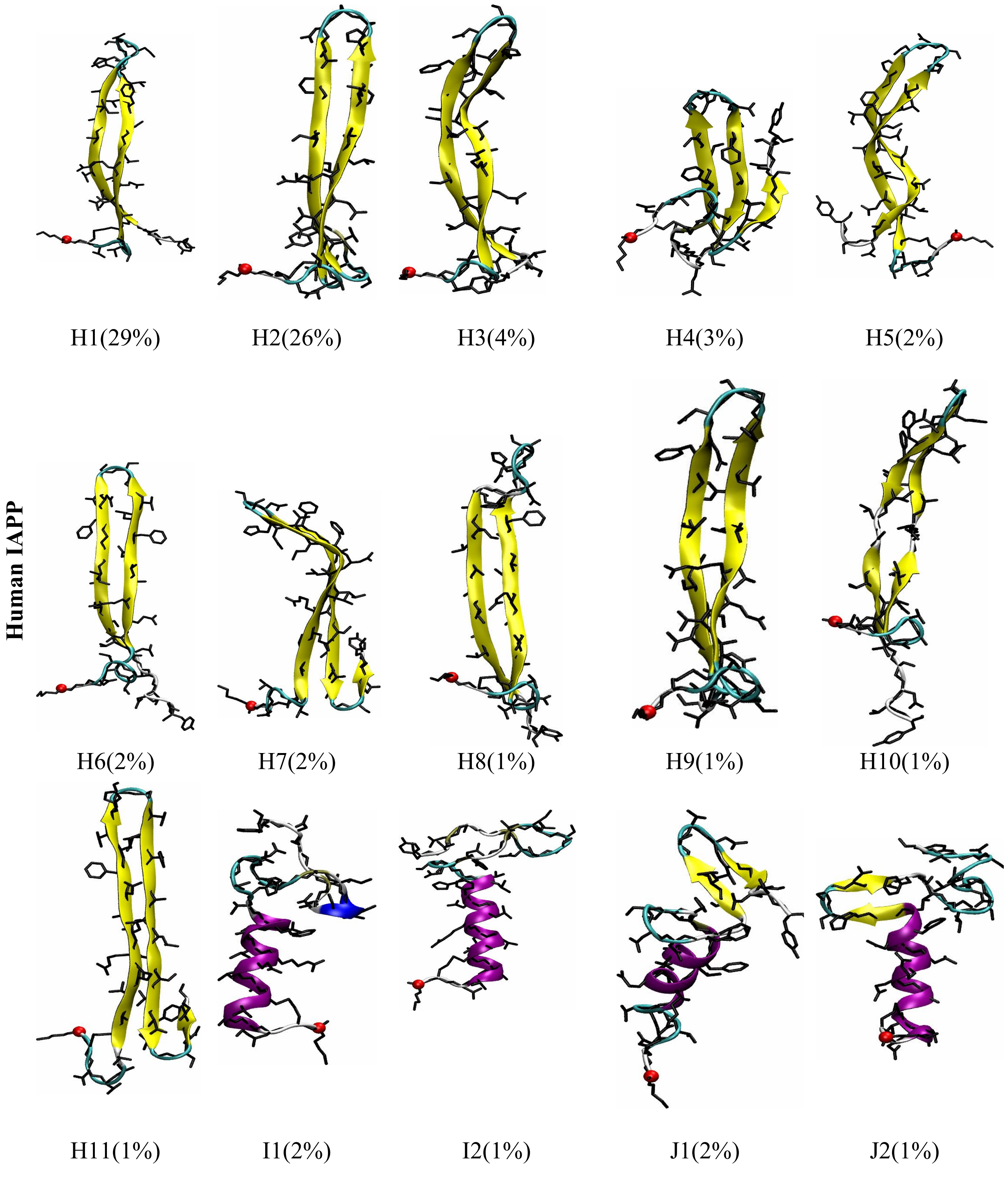
Human IAPP
H1(29%)
H2(26%)
H3(4%)
H4(3%)
H5(2%)
H6(2%)
H7(2%)
H8(1%)
H9(1%)
H10(1%)
H11(1%)
I1(2%)
I2(1%)
J1(2%)
J2(1%)
